# Editorial: Glycans: molecules at the interface of immunity and disease

**DOI:** 10.3389/fimmu.2023.1354723

**Published:** 2024-01-05

**Authors:** Stevan A. Springer, Shoib Sawar Siddiqui

**Affiliations:** ^1^ Biology, University of Prince Edward Island, Charlottetown, PE, Canada; ^2^ Life and Medical Sciences, University of Hertfordshire, Hatfield, United Kingdom

**Keywords:** cancer, glycans, glycobiology, immunity, infection, inflammation, lectins, molecular evolution

Biomolecules are evolved machines. The chemical properties of proteins, lipids and nucleotides are manifestly suited to their essential functions (1). What, then, is the intrinsic function of glycans? Organisms use sugars in such variety; could there be any one role that optimizes the use of glycans’ inherently diverse structures and chemistry? If nucleotide polymers are ‘for’ information storage and proteins are ‘for’ catalysis, we suggest that glycans are for context - they extend and modify biochemical capability. Glycans enhance life’s patterning systems; their finely regulated and context-dependent functions augment processes that require diversity and precision (2).

This Research Topic collects research on immunity and disease, where glycans’ diverse structures and functions contextualize and enact responses to illness and infection. The articles in this Research Topic illustrate glycans’ many biological functions, fine-tuned localization and regulation, diverse structures and synthesis, and varied evolutionary history. Understanding patterns that glycans enact and how these patterns respond to organismal state is central to a complete and nuanced picture of immunity and to targeted disease interventions.

## Origins and diversity

Ultimately, the functions of each glycan and glycan-binding molecule begin with the stories of their evolutionary origin and diversification. Günther and Galuska trace the galectin family of sugar-binding proteins back to its original form, detail the diversification of binding functions, and uncover in jawless vertebrates additional functions lost by other vertebrates. In mammals, galectins regulate immune and inflammatory responses. However, the galectin family originated before the vertebrate immune system. Tracing the early diversification of galectins uncovers some of the functions that existed when the need for immunomodulation arose.

## Regulation and recognition

Glycans shape inward aspects of immunity and disease, where their versatility extends the capacity for coordination and self-recognition. Glycans, glycoproteins, and glycan-binding molecules modulate inflammation through various mechanisms. Zhao et al. review the many pro- and anti-inflammatory responses enacted by fibromodulin, a small leucine-rich proteoglycan. They find effects in wound healing, osteoarthritis, tendinopathy, atherosclerosis, and heart failure. Jin and Zong review the direct effects of glycan molecules on immunomodulation, finding that dysregulation of the glycosaminoglycan hyaluronan is a crucial determinant of renal cell carcinoma progression.

The biochemical flexibility of sugars also extends the co-evolutionary interface, allowing hosts and pathogens to counter each other with molecular patterns that manipulate, mislead, outpace, or obscure. When pathogens mimic host patterns, the adaptive immune system can be a final line of defence separating self from non-self. Goodson et al. show that endogenous immunopeptides presented by the major histocompatibility complex (MHC-II) have extensively remodelled glycans. Clues to the source of the immunopeptide (self or non-self) may exist in these patterns of glycan remodelling.

## Functions and losses of function

Comparisons of similar but independently evolved patterns can inspire new functional inferences. Dhar evaluates patterns of Siglec-8 and co-receptor expression in the brain and finds that Siglec-8 localizes to an intracellular compartment, a pattern also found in the CD33 allele that protects against Alzheimer’s disease (3). The independent origin of these similar localization patterns suggests that losses of function often have tangible consequences for disease progression.

Several articles in this Research Topic explore the consequences of losing glycan-binding functions experimentally. Wei et al. induce a genetic loss of function to evaluate its effect, finding that B4GALT3 knockout mice have enhanced anti-tumour immune responses. Ahmad et al. develop a chemical loss of function. They describe SHG-8, a small molecule that obstructs the binding pocket of Siglec-15, inducing apoptosis in SW480 colorectal cells via changes in microRNA expression. Cao et al. induce loss of function by glycan removal. Desialylation of ligands on eosinophils and mast cells disrupts cis-binding to Siglec-8, allowing them to undergo apoptosis without cytokine priming.

All three of the above studies describe specific interventions that act only in a particular context, highlighting the importance of glycans in targeting interventions. However, Manni et al. describe the presence of Neu5Gc-containing gangliosides in cancer cells and non-cancerous skin cells. Thus, they caution against using these Neu5Gc-glycolipids as targets for cancer immunotherapy. Glycobiology may be a scalpel that divides body-wide phenomena like inflammation and immunity into individually addressable components. Still, those who would intervene must take care to understand the complete pattern.

## Applications

Glycosylation patterns can serve as markers of autoimmune disease because they change in response to physiological state. Lu et al. use a case-control study to show that N-glycosylation of immunoglobulin G (IgG) up-regulates the pro-inflammatory response in lupus nephritis, a disease of chronic inflammation. Wojcik et al. use a longitudinal study to show that patterns of IgG Fc-glycosylation can predict relapses in cases of inflammatory vasculitis.

Glycans in non-immune tissues also change in response to inflammatory disease. Alvarez et al. show that chronic lung inflammation is associated with increased epithelial glycan fucosylation, likely an over-activation of a natural mechanism for recruiting innate immune cells. Pickering et al. use proteomics to discover glycan patterns in blood that can predict whether immune checkpoint inhibitor therapy will be effective in patients with melanoma. In all these cases, glycans are more than just disease markers; their differences intertwine with the causes of the disease. Interpreting such patterns is vital for directing and predicting patient responses to interventions.

## The many futures of glycobiology

Glycans create and enhance biological patterns. They have rich biological functions, and researchers are actively discovering new evolutionary relationships, historical origins, structures, synthesis pathways, cellular localizations, expression modes, and activities (4). Their context-dependent nature has made studying glycans challenging - but there has never been a better time to study biological patterns. Machine learning, modern statistical tools, and genome-scale spatial and temporal data will combine to reveal many formerly intractable features of glycans (5). The determinants of assembly and localization, the space of possible forms, and the grammars of glycan recognition may soon be within reach for many sugars and sugar-binding molecules.

The articles in this Research Topic detail the intricate involvement of glycan functions in immunity and disease. Glycans are often the front line of immunity and the best or only markers for immunological diseases, so the fine context-dependent activities of glycans may also be a means to design precisely targeted interventions. Thus, for both fundamental and practical reasons, the world of biological context and the role of glycans in creating its diverse and distinctive patterns deserve comprehensive exploration.

## Author contributions

SAS: Writing – original draft. SSS: Writing – review & editing.
